# Complete Genome Sequence of *Mycobacterium stephanolepidis*

**DOI:** 10.1128/genomeA.00810-17

**Published:** 2017-08-17

**Authors:** Hanako Fukano, Mitsunori Yoshida, Yukie Katayama, Tsutomu Omatsu, Tetsuya Mizutani, Osamu Kurata, Shinpei Wada, Yoshihiko Hoshino

**Affiliations:** a Department of Mycobacteriology, Leprosy Research Center, National Institute of Infectious Diseases, Tokyo, Japan; b Research and Education Center for Prevention of Global Infectious Diseases of Animals, Tokyo University of Agriculture and Technology, Tokyo, Japan; c The Laboratory of Aquatic Medicine, Nippon Veterinary and Life Science University, Musashino, Japan

## Abstract

*Mycobacterium stephanolepidis* is a rapid-growing nonpigmented species isolated from marine teleost fish (*Stephanolepis cirrhifer*) and is closely related to *Mycobacterium chelonae*. Here, we report the complete sequence of its genome, comprising a 4.9-Mb chromosome. The sequence represents essential data for future phylogenetic and comparative genome studies of this fish pathogen.

## GENOME ANNOUNCEMENT

*Mycobacterium stephanolepidis* was recently reported by Fukano et al. (1) as a pathogenic agent in thread-sail filefish (*Stephanolepis cirrhifer*) and black scraper (*Thamnaconus modestus*) (1, 2). Diseased fish had many white nodules on the surface of the serosae of their internal organs and mesentery (1). The first isolate (NJB0901^T^) is a reference strain of *M. stephanolepidis*. Here, we report the complete genome sequence of NJB0901^T^.

The strain was grown on Middlebrook 7H10 agar medium. DNA was purified with the QIAamp DNA minikit (Qiagen, Hilden, Germany) and NucleoSpin plant II kit (Macherey-Nagel, Düren, Germany). The genome sequence was determined using PacBio reads (14,898 reads, 185,224,950 bases) obtained by the RSII system (Menlo Park, CA, USA) and Illumina 300 × 2 paired-end reads (13,282,712 reads) obtained by a MiSeq sequencer (Illumina, San Diego, CA, USA) (3). The PacBio reads were assembled with Canu version 1.5 (4) into two contigs and circularized using Circlator (5). Alignments produced by mapping Illumina reads to the assembly using the Burrows-Wheeler aligner (6) were used for sequence and assembly error correction with Pilon (7).

The chromosome of NJB0901^T^ is 4,994,485 bp in length, with 64.0% G+C content. The average nucleotide identity was 93.56% between *Mycobacterium stephanolepidis* and *M. chelonae* (strain CCUG 47445). The chromosome contains 4,981 predicted protein-coding sequences (CDSs), which is similar to the number of CDSs in *M. chelonae* strain CCUG 47445 (4,921 CDSs). The genome sequence of *M. stephanolepidis* NJB0901^T^ represents essential data for future phylogenetic and comparative genome studies of the *M. chelonae-M. abscessus* group.

### Accession number(s).

This complete genome sequence has been deposited at DDBJ/ENA/GenBank under the GenBank accession no. AP018165.

## References

[1] Fukano H, Wada S, Kurata O, Katayama K, Fujiwara N, Hoshino Y 2017 *Mycobacterium stephanolepidis* sp. nov., a rapid-growing species related to *Mycobacterium chelonae*, isolated from marine teleost fish, *Stephanolepis cirrhifer*. Int J Syst Evol Microbiol, in press. 10.1099/ijsem.0.002028 28857733

[2] Fukano H, Wada S, Kurata O, Mizuno K, Nakanaga K, Hoshino Y 2015 Nontuberculous mycobacteriosis in farmed thread-sail filefish *Stephanolepis cirrhifer*. Fish Pathol 50:68–74. doi:10.3147/jsfp.50.68.

[3] Yoshida M, Nakanaga K, Ogura Y, Toyoda A, Ooka T, Kazumi Y, Mitarai S, Ishii N, Hayashi T, Hoshino Y 2016 Complete genome sequence of *Mycobacterium ulcerans* subsp. *shinshuense*. Genome Announc 4:e01050-16. doi:10.1128/genomeA.01050-16. 27688344 PMC5043562

[4] Koren S, Walenz BP, Berlin K, Miller JR, Bergman NH, Phillippy AM 2017 Canu: scalable and accurate long-read assembly via adaptive k-mer weighting and repeat separation. Genome Res 27:722–736. doi:10.1101/gr.215087.116. 28298431 PMC5411767

[5] Hunt M, Silva ND, Otto TD, Parkhill J, Keane JA, Harris SR 2015 Circlator: automated circularization of genome assemblies using long sequencing reads. Genome Biol 16:294. doi:10.1186/s13059-015-0849-0. 26714481 PMC4699355

[6] Li H, Durbin R 2009 Fast and accurate short read alignment with Burrows-Wheeler transform. Bioinformatics 25:1754–1760. doi:10.1093/bioinformatics/btp324. 19451168 PMC2705234

[7] Walker BJ, Abeel T, Shea T, Priest M, Abouelliel A, Sakthikumar S, Cuomo CA, Zeng Q, Wortman J, Young SK, Earl AM 2014 Pilon: an integrated tool for comprehensive microbial variant detection and genome assembly improvement. PLoS One 9:e112963. doi:10.1371/journal.pone.0112963. 25409509 PMC4237348

